# Low-dose computed tomography for lung cancer screening in Anhui, China: A randomized controlled trial

**DOI:** 10.3389/fonc.2022.1059999

**Published:** 2022-12-14

**Authors:** Feng Rong, Rui Shi, Lili Hu, Ran Chen, Daoyue Wang, Xiazhi Lv, Yong Zhao, Wei Huang, Yang Yang, Hongyang Zhou, Kaige Hong

**Affiliations:** Cancer Centre, Lu’an Hospital of Anhui Medical University, Lu’an, Anhui, China

**Keywords:** female lung cancer, low-dose computed tomography, lung cancer screening, passive smoking, randomized controlled trial

## Abstract

**Background:**

Lung cancer is the leading cause of cancer-related death worldwide, with risk factors such as age and smoking. Low-dose computed tomography screening can reduce lung cancer mortality. However, its effectiveness in Asian populations remains unclear. Most Asian women with lung cancer are non-smokers who have not been screened. We conducted a randomized controlled trial to evaluate the performance of low-dose computed tomography screening in a Chinese population, including high-risk smokers and non-smokers exposed to passive smoking. The baseline data are reported in this study.

**Methods:**

Between May and December 2019, eligible participants were randomized in a ratio of 1:1:1 to a screening (two arms) or control cohort. Non-calcified nodules/masses with a diameter >4 mm on low-dose computed tomography were considered positive findings.

**Results:**

In total, 600 patients (mean age, 59.1 ± 6.9 years) underwent low-dose computed tomography. Women accounted for 31.5% (189/600) of patients; 89.9% (170/189) were non-smokers/passive smokers. At baseline, the incidence of lung cancer was 1.8% (11/600). The incidence of lung cancer was significantly lower in smokers than in female non-smokers/passive smokers (1.0% [4/415] *vs.* 4.1% [7/170], respectively; *P*=0.017). Stage 0–I lung cancer accounted for 90.9% (10/11) of cases.

**Conclusions:**

We demonstrate the importance of including active smokers and female non-smokers/passive smokers in lung cancer screening programs. Further studies are needed to explore the risk factors, and long-term cost–benefit of screening Asian non-smoking women.

**Clinical trial registration:**

http://chictr.org.cn/showproj.aspx?proj=39003, identifier ChiCTR1900023197.

## 1 Introduction

Lung cancer is the leading cause of cancer-related death worldwide (1, 2). Most patients with lung cancer have advanced disease that is incurable. With advances in the understanding of the pathogenesis of lung cancer and the emergence of new treatment strategies, such as targeted therapy and immunotherapy, the long-term outcomes of patients with advanced lung cancer have improved significantly. However, the 5-year survival rate of patients with advanced non-small cell lung cancer was <5% in the chemotherapy era, and is only 13.4% in the immunotherapy era (3).

Early diagnosis and treatment of lung cancer through screening can improve outcomes (4). European and American studies (5–7) have shown that low-dose computed tomography (LDCT) screening reduces the risk of mortality by 20% to 26%. The criteria for selecting high-risk individuals for lung cancer screening in Europe and the United States are based on age and smoking history (4, 8, 9). Nevertheless, the effectiveness of lung cancer screening is influenced by race/ethnicity (10). Studies have shown that lung cancer in Asia differs from that in other regions, in terms of risk factors (10, 11), population characteristics (12), imaging manifestations (13), and screening effectiveness (12, 14). For instance, lung cancer detected by screening in Asia is more radiologically characterized by mixed and pure ground-glass nodules (10, 12). Therefore, the performance of LDCT screening for lung cancer in Asian smokers needs to be clarified.

Lung cancer in women is increasing worldwide (1). However, the increase in lung cancer in Asian women is not fully explained by smoking. In China (2) and South Korea (15), the proportion of female smokers has remained stable at 2.0–6.4%. In Asia, the smoking rate among female lung cancer patients is <20%, while in Europe and the United States it reaches 70–85% (16). Non-smoking lung cancer accounts for the majority of Asian female lung cancers. Unfortunately, these patients are not included in LDCT screening programs. For non-smoking women, the focus is on identifying risk factors in those who may benefit from screening (17). Currently, risk factors for lung cancer in non-smoking Asian women are poorly defined (18). Environmental pollutants, such as environmental tobacco smoke (ETS), particulate matter 2.5, and kitchen fumes, may be risk factors for lung cancer (19). In particular, ETS is associated with the high prevalence of female lung cancer in Asia (19).

China is the largest producer and consumer of tobacco (20); 52.1% of adult males are smokers (2). Women may be more susceptible to ETS at home, in the workplace, and in public places (21, 22). Traditional Chinese patriarchal culture may further increase the risk of ETS exposure at home (21, 23). Passive smoking is an important risk factor for lung cancer in non-smoking Chinese women (24). Better understanding of the screening performance in non-smoking women exposed to passive smoking is needed. Some population-based prospective (14, 25) and retrospective (11) screening studies have included women. However, the risk factors were difficult to analyze, or were not focused on ETS. The ongoing TALENT study focuses on non-smokers but has not yet been peer-reviewed or published. More clinical trial data are needed to elucidate the risk factors, inclusion criteria, and efficacy of LDCT screening of non-smoking women for lung cancer.

We conducted a randomized controlled trial to evaluate the performance of LDCT screening in a Chinese population, including high-risk smokers and non-smokers exposed to passive smoking. The baseline data are reported in this study.

## 2 Materials and methods

### 2.1 Ethics approval

The study was approved by the Ethics Committee of Lu’an Hospital of Anhui Medical University, Anhui, China (2019–002–01). The study was registered in the Chinese Clinical Trial Registry (ChiCTR1900023197). All participants provided written informed consent.

### 2.2 Study design

This was a prospective, randomized controlled trial that was primarily designed to assess the performance of LDCT lung cancer screening in asymptomatic high-risk populations in mainland China. The secondary aim was to explore the clinical value of inflammatory markers in screening for lung cancer.

From May to December 2019, participants were recruited from a community health center and *via* advertisements in 16 communities. At the time of randomization, eligible participants had to be between 50 and 75 years of age, with at least one high-risk factor: (1) A smoking history of ≥30 pack-years (current smoker or former smoker with ≤15 years since quitting); (2) A non-smoker exposed to passive smoking at home (from a family member) or in the workplace (from a colleague) for >20 years; and (3) A non-smoker with a family history of lung cancer. Individuals who had been diagnosed with cancer in the last 5 years, a metallic stent/internal fixation in the chest and/or back, a physical condition rendering them unsuitable for examination, or undergone chest computed tomography in the last 12 months were excluded.

Demographic data and medical histories were recorded at enrollment. Before recruitment, SAS 9.2 software (SAS Institute Inc., Cary, NC, USA) was used to generate random sequences, according to the block randomization method (block=9). To maintain blinding, the sequences were kept by someone who did not understand the random sequence generation method and did not participate in recruitment. Signed informed consent forms were submitted to the sequence custodian, who performed the randomization, according to the randomly assigned sequence.

### 2.3 Screening

Participants were randomized in a ratio of 1:1:1 to three arms: LDCT1 (three rounds of annual LDCT screening), LDCT2 (three rounds of LDCT screening [at baseline, year 1, and year 3] and blood collection) and control (monitoring and questionnaire follow-ups without interfering with normal medical behavior).

Baseline LDCT screening was performed soon after randomization. When the participant was diagnosed with lung cancer, further LDCT screening was not performed. Spiral computed tomography images were obtained using a 32-detector row scanner (Neosoft, China) with a low-dose setting (120 Kvp, 60 mA), and in overlapping contiguous 1-mm increments, with a 1.25 pitch. The images were reviewed by two radiologists with >10 years of experience. The size of the lung nodules/masses, and the maximum diameter in the axial plane, were measured. If there was a disagreement, a chief radiologist specializing in chest imaging was consulted. Positive findings were reviewed by a team consisting of two medical and one thoracic oncologist.

On LDCT, non-calcified nodules with a diameter of >4 mm were regarded as positive results. The average diameter was used for nodules with a longest diameter of <10 mm. The size and radiological features of non-calcified nodules (diameter >4 mm) were recorded. Abnormalities suggestive of clinically significant conditions, other than lung cancer, were also documented, as were minor abnormalities. Screening results were communicated to the participants within 4 weeks. Positive results were communicated within 2 weeks.

### 2.4 Follow-up

The management of positive screening results was carried out according to the China National Lung Cancer screening guideline with LDCT (2018 version) (26). All complications and medical interventions were documented. Participants completed a semiannual Short-Form 36 questionnaire on vital status, with the help of trained interviewers, *via* telephone or in-person interviews. Patients were classified according to the 2015 World Health Organization classification of tumors of the lung and staged according to the eighth edition of the Tumor–Node–Metastasis classification for lung cancer (27). Data were entered into an EpiData database.

### 2.5 Statistical analysis

SPSS for Windows (version 25; SPSS Inc., Chicago, IL, USA) was used for statistical analysis. Continuous variables are presented as mean ± standard deviation or median (interquartile range). Categorical variables are presented as numbers and percentages. Differences in rates between the LDCT and control arms were analyzed using the chi-square or Fisher’s exact test; differences in constituent ratios were analyzed using the Wilcoxon rank sum test. A two-sided *P*-value <0.05 was considered statistically significant.

## 3 Results

### 3.1 Participant characteristics

**Figure 1:**
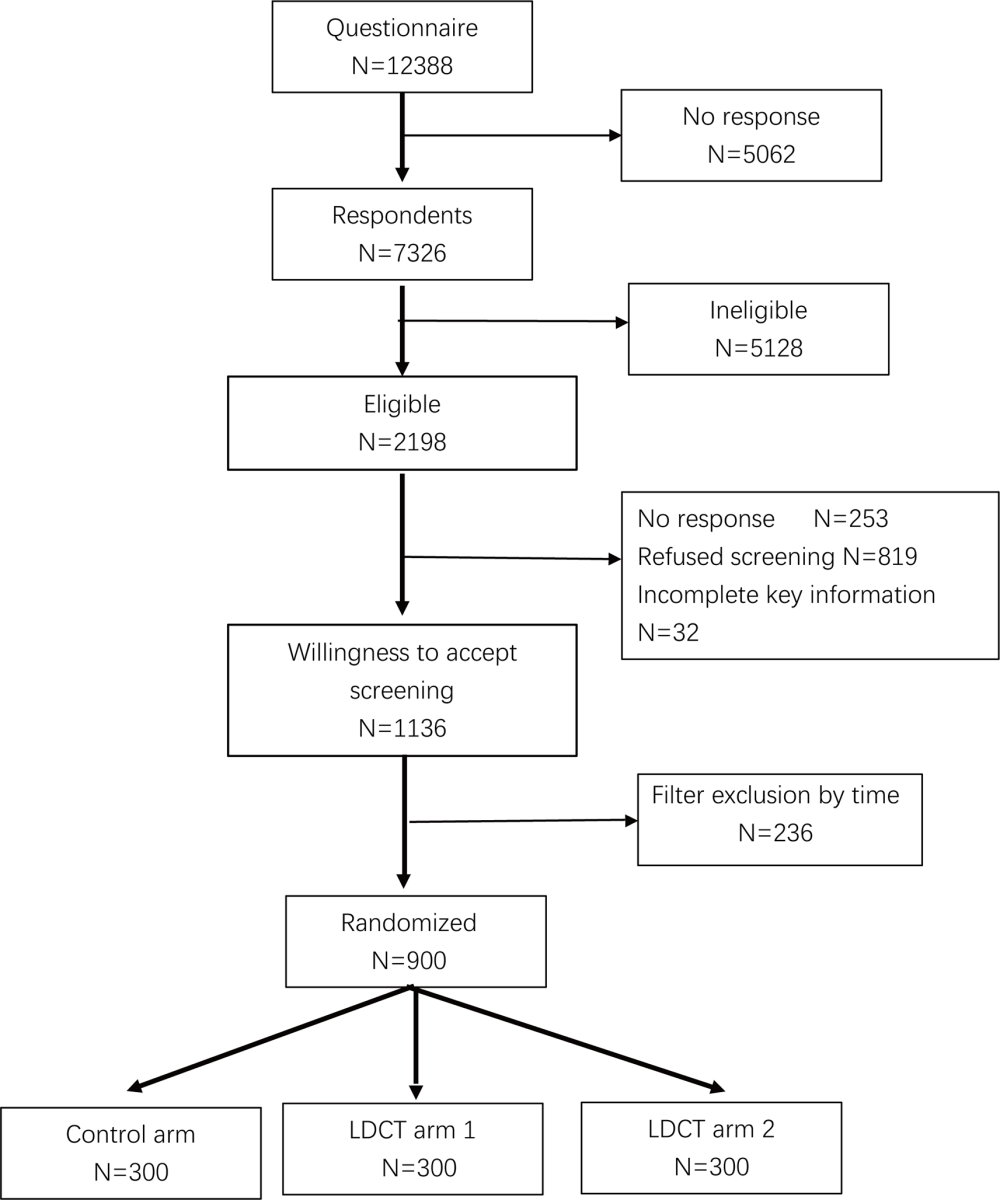
Flowchart of the participants in the trial. LDCT, low-dose computed tomography.

In 16 communities, 12,388 questionnaires were administered. A total of 2,198 of the 7,326 respondents, who returned the questionnaires, were eligible for inclusion. In the second questionnaire, 1,136 participants, who met the inclusion criteria, were willing to undergo LDCT screening. According to the predetermined sample size, the first 900 subjects, who provided written informed consent, were randomly assigned to a screening or control arm (**Figure 1**). As the difference between the LDCT1 and LDCT2 arms mainly lies in the interval between the second and third round of screening, the baseline analysis was a pooled analysis of the two LDCT arms (collectively known as the LDCT group).

There were no significant differences in baseline characteristics between the LDCT and control groups. In the LDCT group (mean age, 59.1 ± 6.9 years), the mean smoking index was 44.7 ± 20.6 pack-years, 32.0% (192/600) of participants were exposed daily to kitchen fumes, and 8.7% (52/600) had a college degree or higher. Women accounted for 31.9% (287/900) of participants; 92.3% (266/287) of women were non-smokers (**Table 1**). All participants met the second criteria (exposed to secondhand smoke at home or in the workplace for >20 years) and were included in the study.

**Table 1:** Participant characteristics according to study group.

Characteristics	LDCT (n=600)	Control (n=300)	*t/X^2^*	*P*-value
Age, years (mean ± SD)	59.1 ± 6.9	59.8 ± 6.9	1.345	0.179
Sex (n)			0.125	0.723
Male	411	202		
Female	189	98		
Smoking history (n)			0.551	0.759
Former	53	23		
Current	362	179		
Passive	185	98		
Smoking index, pack-years (n)			6.423	0.093
30–39	228	91		
40–49	79	50		
50–59	35	24		
>60	73	37		
Tobacco variety (n)			4.956	0.231
Filter	325	149		
Non-filter	90	54		
Hookah	2	2		
Tobacco	0	1		
Cigar	1	0		
Family history of cancer (n)			0.945	0.623
Yes	11	5		
No	440	229		
Unknown	149	66		
Risk factor (n)			0.437	0.804
Kitchen fumes	192	99		
Occupational exposure	34	14		
None	374	187		
Drinking status (n)			0.471	0.790
Former	37	22		
Current	293	143		
Never	270	135		
Education level (n)			5.197	0.074
Primary and below	221	129		
High school	327	155		
College and above	52	16		

LDCT, low-dose computed tomography; SD, standard deviation.

### 3.2 Detection of positive nodules

**Figure 2:**
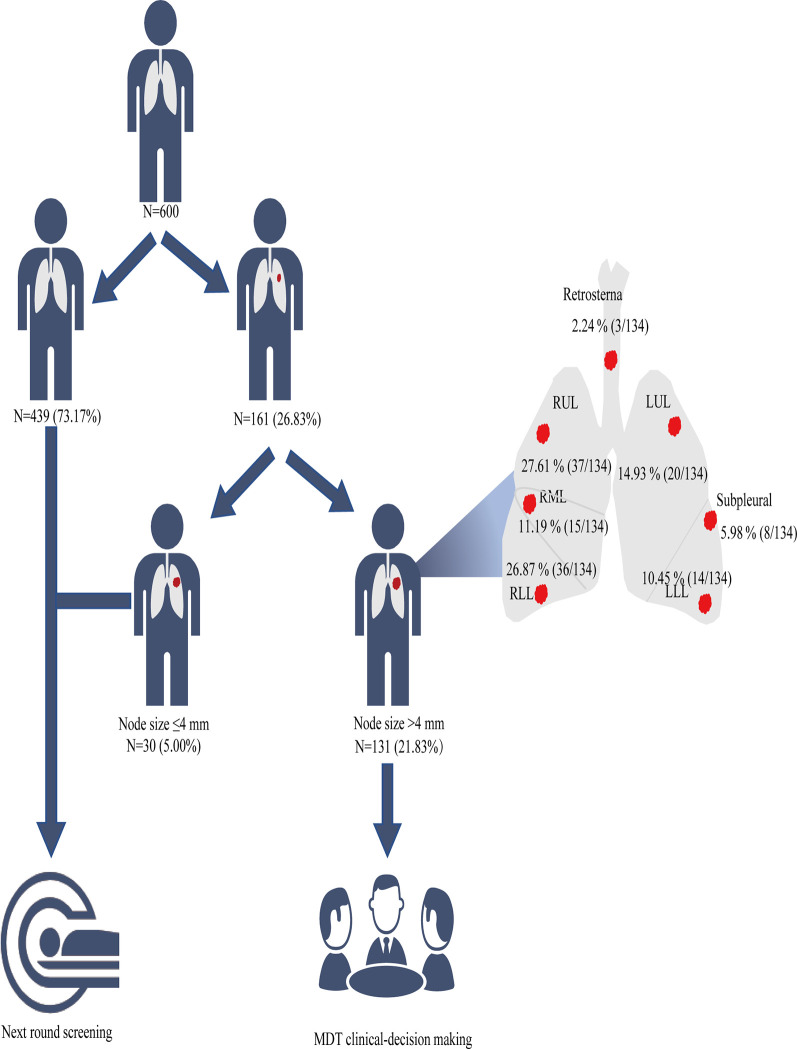
Screening strategy and results of LDCT. LDCT, low-dose computed tomography; LLL, left lower lung; LUL, left upper lung; RLL, right lower lung; RML, right middle lung; RUL, right upper lung.

In the LDCT group, 21.8% (131/600) of participants had lesions measuring >4 mm in the chest, with a total of 134 lesions. There were 130 lesions measuring >4 mm in the lung, three in the posterior thymus, and one in the interlobular fissure. A greater number of positive nodules were located in the right lung (67.8%, 88/130) than in the left lung (26.2%, 34/130) (**Figure 2** and **Table 2**). The proportion of participants with subsolid (mixed and pure ground-glass) nodules was 7.7% (46/600); that in the upper lung was higher than that in the middle and lower lung (69.6% [32/46] *vs*. 6.5% [3/46] and 23.9% [11/46], respectively). (**Table 2**).

**Table 2:** Distribution and type of pulmonary nodules (>4 mm).

Location	Total (n)	Pulmonary nodules, n (%)		
		pGGN	mGGN	SSN
RUL	37	17 (46.0)	3 (8.1)	17 (46.0)
RML	15	3 (20.0)	0 (0.0)	12 (80.0)
RLL	36	7 (19.4)	1 (2.8)	28 (77.8)
LUL	20	11 (55.0)	1 (5.0)	8 (40.0)
LLL	14	1 (7.1)	2 (14.3)	11 (75.6)
Subpleural	8	0 (0.0)	0 (0.0)	8 (100.0)
Total	130	39 (30.0)	7 (5.4)	84 (64.6)

LLL, left lower lung; LUL, left upper lung; mGGN, mixed ground-glass nodule; pGGN, pure ground-glass nodule; RLL, right lower lung; RML, right middle lung; RUL, right upper lung; SSN, solitary solid nodule.

### 3.3 Lung cancer screening

At baseline, the lung cancer detection rate was 1.8% (11/600). All patients had adenocarcinoma. The detection rate of male lung cancer was 0.7% (3/411), which was significantly lower than that of female lung cancer (4.2%, 8/189) (*P*=0.006). Among participants who met the 2021 United States Preventive Services Task Force recommendation on lung cancer screening (4), the lung cancer detection rate was 1.0% (4/415), which was significantly lower than that in female non-smokers (4.1%, 7/170) (*P*=0.017). Stage 0–I lung cancer accounted for 90.9% (10/11) of cases. The proportion of non-smokers was 63.6% (7/11). Female lung cancer accounted for 72.7% (8/11) of cases, of which non-smoking female lung cancer accounted for 87.5% (7/8). There was no significant difference in the diagnosis rate between right and left lung positive nodules (10.2% *vs*. 5.9%, respectively; *P*=0.726) (**Table 3**). None of the participants in the control group were diagnosed with lung cancer in at least one year of follow-up.

**Table 3:** Characteristics of patients diagnosed with lung cancer.

Patient	Sex	Age	Smoking history		Nodule			Pathology		
			Smoking	Pack-years	Size (mm)	Expression	Location	Type	Pathology	Stage
83	F	56	Passive		8.3	mGGN	RUL	AC	IAC	IA
250	M	54	Active	35	9.6	pGGN	LUL	AC	AIS	0
333	F	61	Passive		12.5	mGGN	RUL	AC	IAC	IA
375	F	62	Passive		10.0	mGGN	RUL	AC	AIS	0
430	M	74	Active	86	36.0	SSN (cavity)	LLL	AC	IAC	IA
436	F	52	Passive		9.0	pGGN	RLL	AC	AIS	0
479	F	56	Active	78	6.0	pGGN	RML	AC	MIA	IA
487	F	62	Passive		12.0	pGGN	RUL	AC	MIA	IA
603	M	71	Active	53	48.0	Solid mass	RLL	AC	IAC	III
649	F	52	Passive		5.0	pGGN	RLL	AC	AIS	0
862	F	67	Passive		6.0	pGGN	RLL	AC	AIS	0

AC, adenocarcinoma; AIS, adenocarcinoma *in situ*; F, female; IAC, invasive adenocarcinoma; LLL, left lower lung; LUL, left upper lung; M, male; mGGN, mixed ground-glass nodule; MIA, microinvasive adenocarcinoma; pGGN, pure ground-glass nodule; RLL, right lower lung; RML, right middle lung; RUL, right upper lung; SSN, solitary solid nodule.

## 4 Discussion

In this study, the eligible population included non-smokers other than those who met the 2021 United States Preventive Services Task Force recommendation on lung cancer screening (4). The results showed that the diagnostic rate of lung cancer in female non-smokers was significantly higher than that in smokers. All female non-smokers were passive smokers, suggesting that non-smoking women exposed to passive smoking should be included in Asian high-risk populations for LDCT screening. Our findings add to the evidence regarding the value of LDCT screening in Asia and provide relevant information to optimize and improve the eligibility criteria for LDCT screening in Asian countries.

Age is strongly associated with lung cancer (23). In both men and women, lung cancer is most likely to develop between the ages of 50 and 79 years (28). Mazzone et al. (29) showed that LDCT screening, starting at the age of 50 years and ending at the age of 74–75 years, significantly reduced mortality (hazard ratio: 0.77 [*P*<0.01] and 0.88 [*P*=0.005], respectively). In this study, the inclusion age range was 50–75 years. The mean age was approximately 59 years, similar to those in the NLST (5), NELSON (6), and MILD (30) studies (58–61 years). Taking a diameter of >4 mm as the positive standard, the positive rate of 21.8% was comparable to the T0 rates in the NLST (5), LUSI (7), and UKLS (31) studies (22.2–27.3%). A randomized controlled trial in Shanghai, China (14) showed that the detection rate of non-calcified nodules on initial screening was 22.9%, and that the detection rate of non-calcified nodules measuring ≥5 mm was 13.6%, which were lower than the 26.8% (161/600) and 21.2% (127/600) reported in this study, respectively. This may be attributable to the younger population (45–70 years) in the Yang et al. (14) study. Nevertheless, the incidence of lung nodules increases with age (32).

In the NLST (33) and I-ELCAP (34) studies, subsolid nodules were detected in 9.4% and 4.2% of participants at baseline, respectively. Fan et al. (12) showed that the detection rate of subsolid nodules was no more than 8.6% (1,513 nodules in 17,683 subjects) in Shanghai, China. In this study, subsolid nodules accounted for 7.7% of positive nodules. These findings show that the proportion of subsolid nodules detected by screening is similar in China and the United States and Europe. However, early lung cancer is more likely to present as subsolid nodules in Asia. In this study, subsolid nodules (81.8%) were the main manifestations of lung cancer, consistent with other Asian studies (59.0–84.9%) (10, 12). In Western developed countries, the proportion of subsolid nodules detected by screening was lower (35).

The overall detection rate of lung cancer in this study was 1.8%, which was higher than those in the NLST (1.1%) (36) and NELSON (0.9%) (6) studies, with age and smoking history as inclusion criteria. However, it was similar to screening studies of non-smoking women (10, 13, 37). These differences may be due to the inclusion of non-smokers. In this study, the detection rate of smoking lung cancer was 1.0% (4/415), which was consistent with the aforementioned studies. Similar lung cancer detection rates in Asian smokers further support efforts to include high-risk smokers in screening programs.

In this study, the detection rate of lung cancer in smokers was significantly lower than that in female non-smokers exposed to passive smoking (relative risk: 0.23 [95% confidence interval: 0.07–0.79]; *P*=0.017), consistent with previous studies (10, 37). This difference may be due to the fact that non-smoking female lung cancer patients are more likely to present with ground-glass nodules, and LDCT is advantageous for detecting ground-glass nodules. Non-smoking lung cancer is more common in women, whereas smoking lung cancer does not show significant sex differences (17). This may partially explain the higher detection rate of lung cancer in non-smoking women. The risk factors for lung cancer in Asian women who do not smoke have not been elucidated. However, studies have shown that female non-smoking lung cancer is largely attributed to passive smoking (24, 38). In this study, passive smoking was defined in terms of location (home/workplace) and duration (≥20 years). Forte et al. (18) showed that there was a dose–effect relationship between the duration of exposure to secondhand smoke and lung cancer risk, and that >20 years of passive smoking is the primary determinant of lung cancer risk in women (odds ratio: 1.57 [95% confidence interval: 1.05–2.35]; *P*<0.01). This may also explain the higher detection rate of lung cancer in non-smoking women in this study compared to other studies. We believe that our quantitative measure of passive smoking is more accurate in practice.

Tobacco use is an important mortality factor for lung cancer (39). However, non-smoking lung cancer is the seventh most prevalent malignancy worldwide (17). In Asia, non-smoking lung cancer accounts for the majority of lung cancers in women (16). Therefore, current European and American criteria for selecting eligible patients, based on age and smoking history, may not be applicable to the Chinese population (40), because they may result in a large number of female lung cancer patients being unable to access screening before the onset of symptoms. Abdel-Rahman (41) showed that non-smokers exposed to passive smoking are at a higher risk of developing and dying from lung cancer. Our study also showed that the lung cancer detection rate was higher in non-smoking women exposed to passive smoking. These findings highlight the need to enroll female passive smokers in Asian lung cancer screening programs.

Stage 0–I lung cancer accounted for 90.9% of all lung cancers examined in this study. Overdiagnosis is one focus of lung cancer screening research. Cancers are deemed to have been overdiagnosed if they would not have progressed to a clinical stage throughout the patient’s lifetime (42). Meanwhile, it is the magnitude of overdiagnosis that changes over time as the follow up period is extended. In Asia, overdiagnosis is an issue for lung cancer screening, because more than 90% of lung cancers screened were cStage 0-I. Most of them are indolent. A study (43) from Taiwan showed that LDCT screening in all female nonsmokers may cause a significant overdiagnosis problem. Therefore, age at detection and the definition of risk factors are crucial for lung cancer screening in Asian female nonsmokers.

Although early lung cancer that manifests as ground-glass nodules is usually indolent (44), it may grow rapidly (13), be less stable than that which manifests as mixed ground-glass nodules (45), and require long-term follow-up (46) and treatment (47). We supported the surgical strategies for pre- and minimally invasive lung adenocarcinoma 3.0 proposed by Zhang et al. (48). With the same opinion as Chen (49), we also agree with Detterbeck regarding the observation standards of GGNs by CT. Considering the long lead time of MIA/AIS (50), psychological burden caused by lesions, repeated computed tomography scans, greater surgical resection, and more intensive postoperative surveillance, a multidisciplinary approach is needed.

This study has several limitations. First, it was a single-center study, which may have resulted in selection bias. To avoid bias, we expanded our outreach in the city’s urban areas through on-site and joint outreach with community primary health care providers. Second, passive smoking, exposure to kitchen fumes, and other risk factors have no formal definition and are difficult to assess. In this study, we used the definition of living or working with a smoker for >20 years. Finally, limited by sample size, this study may be not to show an overall survival benefit of screening. However, during COVID-19 pandemic, large-scale clinical researches would meet many challenges. A well-designed study with a small sample size can also provide high-quality data and evidence for future pool analysis.

In conclusion, we conducted a randomized controlled trial to evaluate the performance of LDCT screening in China, using the NLST and customized passive smoking criteria as inclusion criteria. The results confirmed the higher lung cancer detection rate in non-smoking women exposed to passive smoking than in Asian high-risk smokers. We demonstrated the importance of including active smokers and female passive smokers in lung cancer screening programs in China and provided support for the standardization and practice of lung cancer screening in Asia. Further studies are needed to explore the risk factors, and long-term cost–benefit of screening Asian non-smoking women.

## Data availability statement

The original contributions presented in the study are included in the article/supplementary material. Further inquiries can be directed to the corresponding author.

## Ethics statement

The studies involving human participants were reviewed and approved by the ethics committee of Lu’an Hospital of Anhui Medical University. The patients/participants provided their written informed consent to participate in this study.

## Author contributions

FR, YZ, and RS contributed to study design and implementation. FR supervised the study and wrote the first draft of the manuscript. RS, LH, RC, DW, WH, KH, HZ, YY, and XL contributed to the collection and assembly of data. FR, RC, LH, and DW analyzed and interpreted the data. All authors approved the final manuscript and agree to be accountable for all aspects of the work.
